# Achieving a high cure rate with direct‐acting antivirals for chronic Hepatitis C virus infection in Cameroon: a multi‐clinic demonstration project

**DOI:** 10.1111/tmi.13450

**Published:** 2020-07-05

**Authors:** Liza Coyer, Oudou Njoya, Richard Njouom, Tatiana Mossus, Mathurin Pierre Kowo, Frida Essomba, Alexander Boers, Roel Coutinho, Pascale Ondoa, the HEP C‐IMPACT Team, Catherine Bilong, Isabelle Dang Babagnak, Dyane Kamto, Paul Talla, Eric Tchoumi

**Affiliations:** ^1^ Department of Infectious Diseases Public Health Service of Amsterdam Amsterdam The Netherlands; ^2^ Research Laboratory on Viral Hepatitis and Health Communication University of Yaoundé I Yaoundé Cameroon; ^3^ Centre Pasteur du Cameroun Yaoundé Cameroon; ^4^ Joep Lange Institute Amsterdam The Netherlands; ^5^ PharmAccess Foundation Amsterdam The Netherlands; ^6^ Julius Center for Health Sciences and Primary Care University Medical Center Utrecht Utrecht The Netherlands; ^7^ African Society for Laboratory Medicine Addis Ababa Ethiopia; ^8^ Amsterdam Institute for Global Health and Development Amsterdam The Netherlands

**Keywords:** direct‐acting antiviral, Hepatitis C virus (HCV), cure rate, Cameroon, Africa, antiviral à action directe, virus de l'hépatite C (VHC), taux de guérison, Cameroun, Afrique

## Abstract

**Objectives:**

Highly effective direct‐acting antivirals (DAAs) for Hepatitis C treatment are largely inaccessible in sub‐Saharan Africa. Data on treatment feasibility and outcomes in clinical settings are limited. We assessed the feasibility of achieving a high (≥90%) cure rate with DAAs in six gastroenterology clinics in Cameroon.

**Methods:**

Patients with chronic Hepatitis C virus (HCV) infection were treated for 12 or 24 weeks with ledipasvir/sofosbuvir, ledipasvir/sofosbuvir/ribavirin or sofosbuvir/ribavirin, depending on the stage of liver disease and HCV genotype. The cure rate was defined as the proportion of patients with a sustained virological response 12 weeks after treatment completion (SVR12) among all treatment completers.

**Results:**

We identified 190 HCV RNA positive patients between September‐2017 and August‐2018, 161 (84.7%) of whom started treatment. 105 (65.2%) were female, median age was 61.3 years [IQR = 55.9–66.9] and 11 (6.8%) were HIV‐positive. Median plasma HCV RNA was 6.0 log_10 _IU/mL [IQR = 5.6–6.4]. HCV genotypes identified were 1 (34.8%), 2 (13.7%), 4 (50.9%), 1 and 4 (0.6%); 46 (28.6%) strains of 160 single‐genotype infections were non‐subtypeable. Of 158 treatment completers, 152 (96.2%, 95%CI = 91.9–98.6%) achieved SVR12. Six patients did not achieve SVR12: five carried HCV with NS5A resistance mutations and one with NS5B resistance mutations. Three patients died before and two after treatment completion. The most common adverse events were asthenia (12.0%), headache (11.4%) and dizziness (18.9%).

**Conclusion:**

High cure rates of Hepatitis C with DAAs are achievable in clinical settings of Cameroon. However, the accessibility and provision of HCV screening, diagnosis, treatment, monitoring and care should be addressed for large‐scale implementation.

## Abstract (fr)

**Objectifs:**

Les antiviraux à action directe (AAD) hautement efficaces pour le traitement de l'hépatite C sont largement inaccessibles en Afrique subsaharienne. Les données sur la faisabilité du traitement et les résultats en milieu clinique sont limités. Nous avons évalué la faisabilité d'atteindre un taux de guérison élevé (≥90%) avec les AAD dans six cliniques de gastro‐entérologie au Cameroun.

**Méthodes:**

Les patients atteints d'une infection chronique par le virus de l'hépatite C (VHC) ont été traités pendant 12 ou 24 semaines avec le ledipasvir/sofosbuvir, le ledipasvir/sofosbuvir/ribavirine ou le sofosbuvir/ribavirine, selon le stade de la maladie du foie et le génotype du VHC. Le taux de guérison a été défini comme la proportion de patients présentant une réponse virologique soutenue 12 semaines après la fin du traitement (SVR12) parmi tous les patients ayant terminé le traitement.

**Résultats:**

Nous avons identifié 190 patients positifs pour l'ARN du VHC entre septembre 2017 et août 2018, dont 161 (84,7%) ont commencé le traitement. 105 (65,2%) étaient des femmes, l'âge médian était de 61,3 ans [IQR = 55,9‐66,9] et 11 (6,8%) étaient positifs pour le VIH. L'ARN plasmatique médian était de 6,0 log_10_ UI/mL [IQR = 5,6–6,4]. Les génotypes du VHC identifiés étaient 1 (34,8%), 2 (13,7%), 4 (50,9%), 1 et 4 (0,6%); 46 (28,6%) souches provenant de 160 infections à génotype unique n'étaient pas sous‐typables. Sur 158 patients ayant terminé le traitement, 152 (96,2%, IC95%: 91,9–98,6%) ont atteint la RVS12. Six patients n'ont pas atteint la RVS12: cinq portaient le VHC avec des mutations de résistance NS5A et un avec des mutations de résistance NS5B. Trois patients sont décédés avant et deux après la fin du traitement. Les événements indésirables les plus courants étaient l'asthénie (12,0%), les céphalées (11,4%) et les étourdissements (18,9%).

**Conclusion:**

Des taux élevés de guérison de l'hépatite C avec les AAD sont réalisables dans les milieux cliniques du Cameroun. Cependant, l'accessibilité et la fourniture du dépistage, le diagnostic, du traitement, de la surveillance et des soins du VHC devraient être adressés pour une mise en œuvre à grande échelle.

## Introduction

Hepatitis C virus (HCV) infection is a leading cause of liver cirrhosis, hepatocellular carcinoma and liver failure, and estimated to be responsible for almost 500,000 deaths annually [1]. Despite major advances in the development of highly effective direct‐acting antivirals (DAAs) [2, 3], treatment remains largely inaccessible for the vast majority of people living with HCV (PLHCV), especially in low‐ and middle‐income countries (LMICs), mainly due to its associated costs [4, 5].

In 2015, 10.1 of the 71.1 million PLHCV worldwide were estimated to live in sub‐Saharan Africa [6]. HCV is endemic in Cameroon, a lower‐middle‐income country in Central Africa where almost 40% of the 22.7 million inhabitants lived below the national poverty line in 2014 [7]. Results from the 2011 Demographic and Health Survey revealed a 2.5% HCV prevalence (195,000 viremic persons) among adults [8]. Interestingly, local HCV prevalence varied greatly depending on the region, ethnic group and age, reportedly due to historical medical interventions [8, 9, 10, 11, 12].

In Cameroon, the lack of national Hepatitis C screening and treatment guidelines is reflected by a diversity in diagnostic and treatment protocols across care providers, resulting in unnecessary costs and possible sub‐optimal clinical outcomes. Additionally, the lack of national screening and treatment guidelines, central organisation of Hepatitis C care, insufficient technical and human capacity for HCV testing, alongside high costs associated with diagnosis and treatment (generally paid out‐of‐pocket), substantially limit access to HCV diagnosis and care. Most PLHCV in Cameroon are unaware of their status, with a negative impact on transmission and disease progression [13, 14, 15].

To reach the target of 80% treatment coverage set by WHO for 2030, access to affordable and timely treatment and care should be improved alongside the scale‐up of HCV screening [16, 17, 18]. Sharp declines in DAA prices have been achieved in the past years through tiered pricing, which bases prices on a country’s national income, and voluntary licence (VL) agreements, which enable LMICs to purchase generic DAAs from licence holders [4]. While a 12‐week course of branded ledipasvir/sofosbuvir (LDV/SOF) cost $94,500 in the United States [19], this was US$ 840–2040 in countries with VLs in 2017 [4]. In Cameroon, the price for 12 weeks of LDV/SOF was US$ 1203 in 2017, after a government‐negotiated reduction in 2016 [4, 20]. Nevertheless, treatment largely remains prohibitive for many PLHCV in need [21].

The sustainable control of HCV requires the development of a national Hepatitis C treatment programme which further improves financial access to DAAs and laboratory testing, decentralises care and simplifies and standardises diagnostic and treatment protocols. Pay‐for‐performance models of financing may offer an additional solution for improving access to treatment [22, 23]. Their success in a national treatment programme depends on the feasibility of achieving high cure rates in various ranges of clinical settings while reducing the overall costs of treatment.

We assessed the feasibility of achieving a high cure rate using a standardised DAA‐based treatment and diagnostic algorithm, in patients with chronic HCV infection attending six specialised gastroenterology care clinics in Yaoundé, Cameroon.

## Materials and methods

### Study design and sites

HEP C‐IMPACT was a prospective, longitudinal demonstration study to evaluate the feasibility of achieving a high (≥90%) cure rate with DAAs for chronic HCV infection. Six public and private outpatient gastroenterology clinics in Yaoundé, the capital and second largest city of Cameroon, participated in the study. The study applied the negotiated DAA price of the Cameroonian government.

The study was coordinated by the Faculty of Medicine and Biomedical Sciences from the University of Yaoundé. Clinical monitoring was conducted independently by the Agence Nationale de Recherche sur le Sida et les Hépatites virales (ANRS) of Yaoundé. All participants provided written informed consent.

### Patients

Study participants were recruited among patients already identified through gastroenterology consultation. These patients had usually been diagnosed after presenting with liver‐related symptoms or following blood donation [13].

Eligible patients were aged 21 or older and had a positive HCV RNA test. HIV‐positive patients were eligible if they were on antiviral therapy (ART) for ≥ 8 weeks, had undetectable HIV viral load, CD4^+^ T‐cell count of ≥ 350 cells/µL and no signs of opportunistic infections. Patients were excluded if they had (a history of) decompensated cirrhosis (ascites, hepatic encephalopathy or variceal haemorrhage), hepatocellular carcinoma, liver or kidney transplantation, previous DAA treatment failure or current DAA treatment, positive Hepatitis B virus (HBV) surface antigen, renal impairment (creatinine clearance < 50 mL/min as estimated by the Modification of Diet in Renal Disease formula), haemoglobin < 10 g/dL (for those who would be assigned a ribavirin‐based treatment regimen), history of treatment with amiodarone, carbamazepine, phenytoin, phenobarbital, oxcarbazepine, rifabutin, rifampin, rifapentine, St. John’s wort or rosuvastatin, current or anticipated pregnancy and current breastfeeding. Patients with a serious or active medical or psychiatric illness which could interfere with treatment, assessment or adherence and patients deemed incapable of attending follow‐up visits (e.g. living too far from the clinic) were also excluded from the study. Patients who tested negative for HBV surface antigens (HBsAg) and surface antibodies (anti‐HBs) were offered HBV vaccination before starting treatment. Eligibility of potential study participants was discussed during monthly meetings of the College of Gastroenterologists, a technical working group mandated by the Ministry of Health (MoH) to identify patients eligible for DAA treatment.

### Treatment

Treatment options comprised 12 or 24 weeks of a fixed dose of branded LDV/SOF, LDV/SOF with added weight‐based ribavirin (RBV) or SOF/RBV, depending on the stage of liver disease and HCV genotype. Non‐cirrhotic participants with HCV genotype 1 or 4 were offered LDV/SOF for 12 weeks. Cirrhotic participants with HCV genotype 1 or 4 were offered LDV/SOF/RBV (1000 mg of RBV if weight ≤ 75 kg, 1200 mg if> 75 kg) for 12 weeks. Non‐cirrhotic participants with HCV genotype 2 were offered SOF/RBV for 12 weeks, while cirrhotic participants with HCV genotype 2 were offered SOF/RBV for 24 weeks. Cirrhosis was defined as a F4 FibroTest score.

### Procedures

The study was approved by the Cameroon National Ethics Committee (2017/05/913/CE/CNERSH/SP) and the MoH. All study staff signed a confidentiality agreement. Clinicians and study nurses were trained at study inception for good clinical practice. Participants were asked to contribute a quarter of the drug costs (approximately €120–245 depending on treatment regimen, Table S1 of the Appendix S1) upon recommendation from the MoH. All laboratory investigations and consultations were free of charge.

The pre‐enrolment procedures consisted of a standardised liver ultrasound to assess the presence of decompensated cirrhosis, a physical examination and blood sample collection for laboratory investigations. Eligibility criteria were subsequently confirmed at enrolment, after which patients started treatment. Enrolment and follow‐up visits took place at weeks 0, 4, 8, 12 and 24 (12 weeks after completing treatment), with additional week 16, 20 and/or 24 visits for participants with an indication for extended treatment schedules. At enrolment and each follow‐up visit, participants underwent a physical examination, blood draw and counselling for treatment adherence (also for ART if applicable), family planning and prevention of HCV re‐infection. They received a compensation for travel expenses, paid their share of the treatment costs and were given a prescription to collect a 4‐week drugs supply at the pharmacy. Drugs were stored at the central pharmacy of the University Teaching Hospital of Yaoundé at room temperature (≤25^o^C). Participants received a text message every seven days between study visits reminding them to take their medication. Patients were contacted by phone in case of not showing up for a follow‐up visit.

Blood samples collected at each clinic were transported for testing and storage to the Centre Pasteur du Cameroun (CPC) within six hours in a temperature‐controlled box. Phlebotomists were trained to follow the CPC standard operating procedure for sample collection and storage.

### Laboratory testing

All tests except the FibroTest‐ActiTest (done at Cerba Laboratory in France) were done at CPC. An overview of all laboratory examinations per visit is provided in Table S2 of the Appendix S1. In short, pre‐enrolment laboratory investigations comprised testing for plasma HCV RNA levels, stage of liver disease (FibroTest‐ActiTest), HCV genotype (if not already available from patient files), HIV serology, HBsAg, anti‐HBs, creatinine levels, alanine aminotransferase, aspartate aminotransferase, albumin, bilirubin and full blood counts (including haemoglobin concentrations). In addition, CD4^+^ T‐cell counts and plasma HIV RNA levels were determined for HIV‐positive participants and urine β‐hCG levels were measured among female participants of child‐bearing age. HIV serology, urine β‐hCG levels (for women of child‐bearing age) and creatinine levels (for HIV‐positive participants receiving tenofovir) were determined at all follow‐up visits. At the last post‐treatment visit, we also determined plasma HCV RNA and plasma HIV RNA levels (for HIV‐positive participants). Available pre‐ and post‐treatment samples from participants who did not achieve a sustained HCV virological response were analysed for HCV drug resistance mutations. We also retrospectively determined plasma HCV RNA levels at all study visits from these participants using stored plasma samples to confirm a possible viral rebound.

### Virology testing

Plasma HCV RNA amount was measured using the Abbott RealTime HCV assay with a lower limit of detection of 12 IU/mL (Abbott Molecular, Wiesbaden, Germany). Detailed methods for HCV RNA extraction, reverse transcription, amplification, genotyping and sequencing have been described elsewhere [24,25]. Briefly, HCV RNA was extracted from 140µL of plasma using QIAamp Viral RNA Mini Kit (Qiagen, Courtaboeuf, France). For genotyping, core (400 nt fragment) and NS5B (382 nt fragment) regions were amplified using nested polymerase chain reaction (nested‐RT PCR) and sequenced by the Sanger method using GenomeLab DTCS‐Quick Start Kit (Beckman Coulter, Paris, France). Nucleotide sequences were subsequently compared to reference sequences representing HCV genotypes and subtypes from Genbank [26] using ClustalW to determine HCV genotype and subtype. For the identification of resistance mutations, two segments of the HCV genome including either NS5A and NS5B genes were amplified using combined reverse transcriptase (RT‐PCR) with the one‐step RT‐PCR kit (Superscript III Platinum One‐Step qRT‐PCR system). NS5A and NS5B mutations spectrum were then characterised using the Molecular Evolutionary Genetics Analysis 6 program [27]. HIV serology was conducted using the Abbott Architect HIV Ag/Ab Combo assay (Abbott Combo, Abbott Laboratories, Abbott Park, IL, USA) and the Genscreen HIV 1/2 version 2 test (Bio‐Rad Laboratories, France). Plasma HIV RNA amount was measured using the Abbott RealTime HIV‐1 assay with a lower limit of detection of 40 copies/mL (Abbott Molecular, Wiesbaden, Germany).

### Data collection

All data were collected using an electronic case report form (e‐CRF) on tablets [28]. Demographic and socio‐economic characteristics, lifetime HCV risk factors, clinical status and laboratory test results were collected at pre‐enrolment. Information on treatment adherence and laboratory test results was collected at each follow‐up visit and entered into the e‐CRF (for details see the Appendix S1).

We collected data on financial hardship using the Poverty Probability Index (PPI) of Cameroon, a 10‐item questionnaire including questions on a household’s characteristics and assets to estimate the probability of living below the national poverty line [29].

### Outcome measures

The primary outcome was the proportion of participants with a sustained virological response, defined as undetectable plasma HCV RNA 12 weeks after treatment completion (SVR12), among all treatment completers (cure rate), even when assigned regimen deviated from the protocol. This was intended to reflect ‘real‐world’ clinical practice.

Secondary outcomes were detection of drug resistance mutations among those who did not achieve SVR12, treatment adherence, adverse effects and delays in treatment and care. We also calculated the costs of treatment and diagnostics per participant. Treatment adherence was measured using the six‐item Test for Evaluating Observance (TEO) and categorised into good (TEO = 0), suboptimal (TEO = 1 or 2) and insufficient (TEO ≥ 3) [30]. We calculated (i) time between pre‐enrolment and enrolment, (ii) time between enrolment and initial treatment initiation and (iii) treatment interruptions. A treatment interruption was defined as not having enough pills during follow‐up (and did not account for missed doses due to adherence issues), assuming that participants would obtain medication for 28 days at each visit.

### Sample size calculation

Assuming an absolute precision of < 5% between the lower and upper bound of the 95% confidence interval (CI) for an assumed SVR12 of ≥ 90%, a total of 150 participants was necessary.

### Statistical analysis

We calculated the SVR12 and the corresponding 95% CI using the exact method. We compared socio‐demographic characteristics between patients who were included and excluded from the study. We compared treatment adherence at each follow‐up visit between those achieving and not achieving SVR12. Comparisons between groups were done using Pearson's chi‐square, Wilcoxon rank‐sum or Fisher’s exact tests as appropriate. We cleaned and analysed data using Stata/MP 16.0 (StataCorp, College Station, Texas, USA).

## Results

### Cohort profile

**Figure 1:**
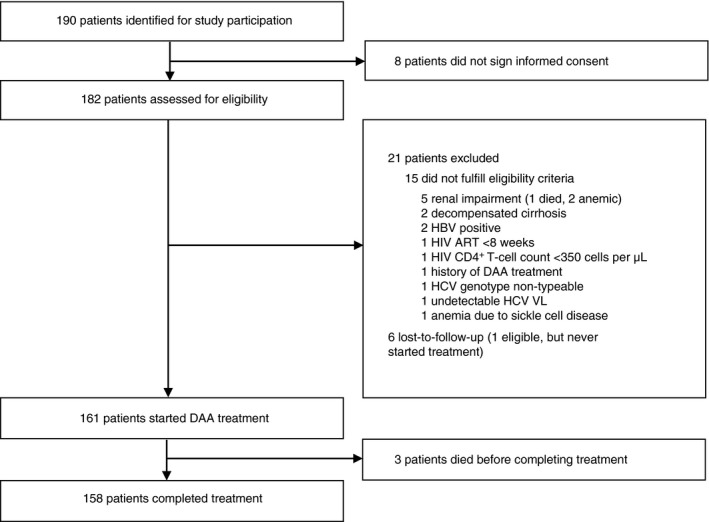
Flow chart of study participation. Abbreviations: ART = antiretroviral therapy, DAA = direct‐acting antiviral, HBV = hepatitis B virus, HCV = hepatitis C virus; HIV = human immunodeficiency virus; SVR12 = sustained virological response 12 weeks post‐treatment

Between September‐2017 and August‐2018, 190 HCV RNA positive patients were identified for study participation, of whom 182 (95.8%) signed informed consent and were assessed for eligibility (Figure 1). Of these, 161 (88.5%) started treatment, 15 (8.2%) were not eligible for inclusion and six (3.3%) were lost to follow‐up (LTFU) before treatment could be started. The most commonly reported exclusion criteria were renal impairment (*n* = 5), decompensated cirrhosis (*n* = 2) and HBsAg positivity (*n* = 2). Among those who started treatment, median time between pre‐enrolment and enrolment was 28 days (IQR 21–40). Thirty (18.6%) participants had a delay between enrolment and treatment initiation (median delay: 5 days [IQR 2–7]). Delays were reportedly due to needing more time to collect enough funds to pay for treatment or unavailability of drugs. All other participants initiated treatment on the same day as enrolment.

In total, 158 (98.1%) of 161 participants who started treatment, completed it. Three participants died before completing treatment. There were no LTFU or new HIV diagnoses during follow‐up. Ten participants interrupted their treatment once and one participant had two interruptions during follow‐up (median interruption length: 2 days [range 1–7]).

### Participant characteristics

Of 161 participants who started treatment, 105 (65.2%) were female (Table 1). Median age was 61.3 years (IQR 55.9–66.9). Median plasma HCV RNA was 6.0 log_10_ IU/mL (IQR 5.6–6.4). Fifty (31.1%) participants had cirrhosis and 11 (6.8%) participants were HIV‐positive, all of whom knew their status. Sixty‐four (39.8%) had a job, while median PPI score among all participants was 66 (IQR 59–74). No differences were found in terms of sex, area of residence, marital status and employment status between those included and those not included in the study (results not shown). The most commonly reported lifetime risk factor for HCV acquisition was a history of collective vaccination (*n* = 120/160, 75.0%) (Table 2). Regular alcohol consumption was reported by 24 (14.9%) participants. Twenty‐nine (18.0%) participants were diabetic and 68 (42.2%) had blood hypertension.

**Table 1:** Socio‐demographic and clinical characteristics of study participants at pre‐enrolment

	Total (* n* = 161)
Clinic	
University Teaching Hospital of Yaoundé	73 (45.3%)
Essos Medical Center	11 (6.8%)
Military Hospital of Yaoundé	8 (5.0%)
Cathedrale Medical Center	22 (13.7%)
Central Hospital of Yaoundé	19 (11.8%)
General Hospital of Yaoundé	28 (17.4%)
Socio‐demographic characteristics	
Age (median, [IQR])	61.3 [55.9–66.9]
Female sex	105 (65.2%)
Living in Yaoundé	154 (95.7%)
Marital status	
Married	95 (59.0%)
Widowed	42 (26.1%)
Single	20 (12.4%)
Divorced	4 (2.5%)
Employment status	
Civil servant	28 (17.4%)
Employed in private sector	17 (10.6%)
Self‐employed	19 (11.8%)
Unemployed	80 (49.7%)
Other	17 (10.6%)
Poverty Probability Index Cameroon (median, [IQR])†	66 [59–74]
Clinical characteristics	
HIV‐positive	11 (6.8%)
CD4 cell count if HIV‐positive (median, [range])	645 [385–1260]
ART regimen if HIV‐positive	
TDF/3TC/EFV	4 (36.4%)
AZT/3TC/NVP	1 (9.1%)
TDF/3TC/NVP	1 (9.1%)
TDF/3TC/ATV	4 (36.4%)
ABC/3TC/EFV	1 (9.1%)
HCV genotype and subtype distribution per genotype	
Genotype 1	56 (34.8%)
1a	4 (7.1%)
1b	14 (25.0%)
1e	13 (23.2%)
1h	2 (3.6%)
1l	7 (12.5%)
1 subtype nontypeable	15 (26.8%)
1 subtype unknown	1 (1.8%)
Genotype 2	22 (13.7%)
2a	1 (4.5%)
2f	2 (9.1%)
2a/c	4 (18.2%)
2 subtype nontypeable	15 (68.2%)
Genotype 4	82 (50.9%)
4e	1 (1.2%)
4f	50 (60.9%)
4p	6 (7.3%)
4t	3 (3.7%)
4a/e	1 (1.2%)
4a/c/d	4 (4.9%)
4 subtype nontypeable	16 (19.5%)
4 subtype unknown	1 (1.2%)
Genotypes 1 and 4	1 (0.6%)
1e and 4 subtype nontypeable	1 (100%)
Median HCV RNA (log_10_ IU/mL [IQR])‡	6.0 [5.6–6.4]
Median aspartate aminotransferase (IU/mL [IQR])	44 [34–61]
Median alanine aminotransferase (IU/mL [IQR])	46 [35–63]
Albumin < 3.5 g/dL	0 (0%)
Platelet count < 90 000 per µL	4 (2.5%)
Aspartate aminotransferase‐to‐platelet ratio index	
≤1.0	128 (79.5%)
>1.0 to ≤ 2.0	19 (11.8%)
>2.0	14 (8.7%)
FibroTest score	
F0–F2	82 (50.9%)
F3	29 (18.0%)
F4	50 (31.1%)
ActiTest score	
A0–A1	101 (62.7%)
A2	33 (20.5%)
A3	27 (16.8%)

ABC, abacavir; ART, antiretroviral therapy; AZT, zidovudine; EFV, efavirenz; HCV, hepatitis C virus; HIV, human immunodeficiency virus; IQR, interquartile range; IU, international units; NVP, nevirapine; RNA, ribonucleic acid; TDF, tenofovir disoproxil fumarate; 3TC, lamivudine.

† 28 missing.

‡ 1 missing.

**Table 2:** Lifetime presence of reported risk factors for acquisition of HCV and severity of liver disease

	Total (*n* = 161)
Transmission risk factors	
Blood transfusion†	32 (20.0%)
Invasive surgery†	70 (43.8%)
Scarring or tattoos	97 (60.3%)
History of collective vaccination†	120 (75.0%)
Injecting drug use†	0 (0%)
Contact with HCV‐positive persons in daily life‡	23 (15.6%)
Traditional treatments with sharp objects†	59 (36.9%)
Other risk factors	6 (3.7%)
Risk factors for severity	
Regular consumption of alcohol, among those with regular consumption:	24 (14.9%)
<14 units of alcohol per week	15 (62.5%)
≥14 units of alcohol per week	9 (37.5%)
Co‐morbidities other than HIV (not mutually exclusive)	
None	68 (42.2%)
Diabetes	29 (18.0%)
Hypertension	68 (42.2%)
Other	20 (12.4%)

Missings were not included in the calculation of percentages. HCV, hepatitis C virus, HIV, human immunodeficiency virus.

† 1 missing.

‡ 13 missing.

### HCV genotypes

HCV genotypes identified were 1 (*n* = 56, 34.8%), 2 (*n* = 22, 13.7%), 4 (*n* = 82, 50.9%), 1 and 4 (*n* = 1, 0.6%) (Table 1). Among the 160 participants with a HCV single‐genotype infection, 46 (28.6%) strains could not be subtyped: 26.8% of genotype 1, 68.2% ofgenotype 2 and 19.5% ofgenotype 4. The predominant HCV single‐genotype 1 subtypes were 1b (*n* = 14, 25.0%) and 1e (*n* = 13, 23.2%). The predominant HCV single‐genotype 4 subtype was 4f (*n* = 50, 60.9%).

### Treatment outcomes

Of the 161 participants initiating treatment, 147 (91.3%) did according to protocol. Of these 147, 90 (61.7%) were treated with LDV/SOF for 12 weeks, 37 (25.2%) with LDV/SOF/RBV for 12 weeks, 19 (12.9%) with SOF/RBV for 12 weeks and one (0.7%) with SOF/RBV for 24 weeks according to protocol. Fourteen (8.7%) participants received a treatment that was not according to protocol: four received LDV/SOF/ for 12 weeks, two received LDV/SOF/RBV for 12 weeks, one received SOF/RBV for 12 weeks, and seven received an alternative treatment regimen (*n* = 6 LDV/SOF for 24 weeks, *n* = 1 LDV/SOF/RBV for 16 weeks). In five cases, the protocol diversion was reportedly motivated by a suspicion of cirrhosis based on discordant FibroTest scores from patient files and at pre‐enrolment. The motivation for initiating an alternative treatment was not communicated for the other nine cases.

Of 158 treatment completers, 152 achieved SVR12 (96.2%, 95% CI 91.9–98.6%). All participants who did not receive treatment according to protocol (*n* = 14) or experienced treatment interruptions (*n* = 11) achieved SVR12. More than 75% of treatment completers reported good adherence at any time point during follow‐up (Table 3). There were no statistically significant differences in adherence between those who achieved SVR12 and those who did not (Table 3).

**Table 3:** Treatment adherence during follow‐up among those who completed treatment

	Total (*n* = 158)	SVR12 (*n* = 152)	No SVR12 (*n* = 6)	Fisher’s exact test *P*‐value
Week 4				
Good adherence	123 (77.9%)	119 (78.3%)	4 (66.7%)	0.62
Suboptimal adherence	35 (22.2%)	33 (21.7%)	2 (33.3%)	
Insufficient adherence	0 (0%)	0 (0%)	0 (0%)	
Week 8				
Good adherence	123 (77.9%)	119 (78.3%)	4 (66.7%)	0.62
Suboptimal adherence	35 (22.2%)	33 (21.7%)	2 (33.3%)	
Insufficient adherence	0 (0%)	0 (0%)	0 (0%)	
Week 12				
Good adherence	125 (79.1%)	121 (79.6%)	4 (66.7%)	0.61
Suboptimal adherence	33 (20.9%)	31 (20.4%)	2 (33.3%)	
Insufficient adherence	0 (0%)	0 (0%)	0 (0%)	

Good adherence was defined as a TEO score of 0, suboptimal adherence as a TEO of 1 or 2 and insufficient adherence as a TEO of 3 or more. SVR, sustained virological response; TEO, Test for Evaluating Observance.

### Treatment failures

Six participants did not achieve SVR12. All were HIV‐negative and experienced a HCV relapse (i.e. had undetectable HCV RNA at week 12, but detectable 12 weeks post‐treatment as confirmed by retrospective HCV VL testing of plasma) (Table 4). Two reported suboptimal adherence at weeks 4, 8 and 12. NS5A resistance mutations were identified on genome positions 24 (*n* = 3), 28 (*n* = 5), 30 (*n* = 4) and 31 (*n* = 5) in five participants; sequencing failed for one participant. These mutations were identified in both the pre‐ and post‐treatment samples for four participants, whereas no pre‐treatment sample was available for the fifth. Only post‐treatment samples were available for sequencing of the NS5B region. NS5B resistance mutations were identified on genome positions 282 and 316 in one participant. No NS5B resistance mutations were identified in the other five.

**Table 4:** Characteristics and virological outcomes of participants who did not achieve sustained virological response

Participant	HIV status	HCV genotype and subtype	Treatment	HCV RNA (log_10_ IU/mL)			Adherence	Sample used for sequencing	NS5A resistance mutations	NS5B resistance mutations
				Week 0	Week 2	Week 12 PT				
1	Negative	1e	LDV/SOF 12 weeks	4.5	undetectable	6.5	Good adherence	Post‐treatment	K24G, L28M, R30Q, L31M	None identified
2	Negative	4f	LDV/SOF 12 weeks	6.1	undetectable	5.8	Good adherence	Pre‐treatment	L28M, L31M	Not sequenced
								Post‐treatment	L28M, L31M	S282T, C316N
3	Negative	1l	LDV/SOF 12 weeks	6.4	undetectable	6.5	Good adherence	Pre‐treatment	K24G, L28M, R30Q, L31M	Not sequenced
								Post‐treatment	K24G, L28M, R30Q, L31M	None identified
4	Negative	1nc	LDV/SOF 12 weeks	6.9	undetectable	5.8	Suboptimal adherence	Pre‐treatment	L28M, R30Q, L31M	Not sequenced
								Post‐treatment	L28M, R30Q, L31M	None identified
5	Negative	1l	LDV/SOF/RBV 12 weeks	6.6	undetectable	5.8	Suboptimal adherence	Pre‐treatment	K24S, L28M, R30Q, L31M	Not sequenced
								Post‐treatment	K24S, L28M, R30Q, L31M	None identified
6	Negative	4nc	LDV/SOF 12 weeks	6.1	undetectable	6.3	Good adherence	Post‐treatment	Sequencing failed	None identified

LDV, ledipasvir; HIV, human immunodeficiency virus; IU, international unit; SOF, sofosbuvir; RBV, ribavirin; RNA, ribonucleic acid.

### Adverse events

Eighty‐seven (54.0%) of 158 treatment completers experienced a minor adverse event. The three most common adverse events were asthenia (*n* = 19, 12.0%), headache (*n* = 18, 11.4%) and dizziness (*n* = 14, 18.9%) (Table 5).

**Table 5:** Most common adverse events (>5%) among those who completed treatment

	Total (*n* = 158)
Asthenia	19 (12.0%)
Headache	18 (11.4%)
Dizziness	14 (8.9%)
Fatigue	9 (5.7%)
Insomnia	9 (5.7%)
Flu symptoms	9 (5.7%)
Cough	9 (5.7%)
Back pain	9 (5.7%)
Cramp	9 (5.7%)
Epigastric pain	8 (5.1%)
Malaria	8 (5.1%)

Two of the three participants who died during treatment had been incorrectly included in the study despite renal impairment. Both participants were HIV‐positive and had been hospitalised during follow‐up prior to their death. One was a 59‐year‐old female who died at home shortly after her week 8 visit after hospital discharge. The other was a 55‐year‐old male with diabetes, hypertension and cirrhosis, who died in the hospital around week 12 of the study. The third participant was a 74‐year‐old, HIV‐negative female with diabetes, hypertension and cirrhosis. She suffered from a stroke within four weeks after starting treatment and died at home in week 8 of the study. The participants who died after treatment completion were HIV‐negative males, of, respectively, 75 and 80 years old and had diabetes, hypertension and cirrhosis.

### Treatment costs

Based on applied prices of drugs and tests and the costs incurred for coordination, supervision and consultation within the programme, the total cost of Hepatitis C management amounted to €1914.42 (US$ 2105.71) per participant, with the diagnostic costs being 40% higher than the treatment costs (€663.91 vs. €477.75 for 12 weeks of LDV/SOF) (Tables S1‐S3 of the Appendix S1). Potential opportunities for further cost reduction exist and are listed in the Appendix S1. A reduction of €293.91 per participant could be achieved by using pan‐genotypic DAAs to avoid the need for HCV genotyping, adopting the APRI and/or FIB‐4 score instead of the FibroTest and reducing the number of ultrasound specialised consultation. Further reducing the number of consultations and shifting tasks to non‐specialised healthcare providers offer additional opportunities for cost reduction while promoting decentralisation of care.

## Discussion

Here, we report a 96.2% cure rate of Hepatitis C with DAAs, thereby demonstrating that achieving a cure rate comparable to those observed in high‐income countries is feasible in a lower‐middle‐income African country when specialised gastroenterology care is provided according to a standardised protocol and at relatively reduced costs. Our results indicate that high cure rates with DAAs can be achieved, regardless of HCV genotype and including HIV‐positive patients, in contrast to the much lower rates previously observed for treatment with pegylated interferon alpha‐2a [31, 32]. Our data corroborate other reports from Senegal, Côte d’Ivoire, Cameroon and Ethiopia [33, 34, 35, 36].

Our high cure rate was achieved in the context of high levels of treatment adherence, few delays in treatment initiation, rare cases of treatment interruptions, and no LTFU. The close supervision of the study nurses, who used a detailed e‐CRF and sent reminders for follow‐up visits, may have contributed to these positive outcomes. Our results show that the protocol was easy to follow, though a couple of protocol violations were observed. While practice should allow some flexibility in treatment assignment as based on informed decisions of clinicians, guidelines should be strong, clear and simple, and training for compliance should be provided.

The general health of PLHCV in this cohort was poor, likely due to late diagnosis after the onset of liver disease symptoms and in the context of high age and presence of other co‐morbidities [13]. Kidney failure and chronic diseases such as diabetes and hypertension are prevalent among older PLHCV with a higher risk of mortality [37, 38]. This is consistent with observations from this study. Median age of participants was 62 years, 18% had diabetes, 42% hypertension and 31% cirrhosis. Multiple co‐morbidities were also present among those who died during and after treatment. Alongside monitoring liver disease progression, future treatments programmes should therefore consider the management of other health needs and associated costs, and avoid suboptimal treatment outcomes or adherence issues possibly arising from a high pill burden [39].

The NS5A and NS5B mutations identified on several positions of the genome in five of the six patients not achieving SVR12 are known to confer resistance to ledipasvir and sofosbuvir, respectively, albeit from studies conducted in Europe and North America [40]. The preferred drug of choice for these patients would be sofosbuvir/velpatasvir/voxilaprevir. Confirming the actual contribution of these mutations to the treatment failures requires additional analyses to compare the prevalence of the mutations between those achieving and not achieving SVR12. Further full‐length sequencing is warranted to characterise the quarter of HCV strains that could not be subtyped in our study.

Challenges with access to HCV screening, treatment and care compromise the achievement of HCV elimination despite the feasibility of high cure rates. Patients included in this study had already been diagnosed and linked to specialised care, thereby representing only a minority of PLHCV in Cameroon. Only 20% of PLHCV worldwide had been diagnosed in 2016, with even fewer accessing treatment [4]. Diagnosis and clinical management of HCV is hampered by the unavailability of affordable and simple diagnostic tests and unnecessarily complicated diagnostic algorithms [14]. A reduction and simplification of these algorithms, including alternatives for diagnostics currently used, is warranted to reduce costs and decentralise testing. Here, we show that providing Hepatitis C treatment based on only two HCV viral loads (instead of the four in current practice in Cameroon) is safe and feasible. Further options for cost reductions are possible including the transition to pan‐genotypic treatment [41, 42] and selection of more affordable options to grade the stage of liver disease. Additionally, molecular point‐of‐care testing platforms such as GeneXpert [43] and Genedrive [44] for viral load quantification could facilitate decentralised viral load testing, provided that necessary quality assurance systems are put in place.

Current negotiated DAA prices still surpass the median annual income per capita and are unaffordable for the annual health budgets of the majority of African countries, which are burdened by other infectious diseases, thereby preventing the scale‐up of public health treatment programmes [21]. All participants in this study appeared to be relatively wealthy, based on high PPI scores and as confirmed by their ability to pay a quarter of the drug costs. However, the majority of PLHCV in Cameroon are anticipated to be unable to afford the out‐of‐pocket costs for treatment and care in the absence of support from a donor or national treatment programme. The high cure rate obtained in this study confirms that alternative innovative financial strategies, such as pay‐for‐performance mechanisms, are feasible [22, 23, 45]. These types of models will require further improvements to the cost‐effectiveness of treatment and eliminations of remaining programmatic inefficiencies that result in the waste of resources.

Improvements to the accessibility and quality of HCV care needs to happen in conjunction with cost reductions to increase the impact of a national treatment programme. HCV care in Cameroon is currently organised centrally around specialised gastroenterology clinics. Decentralisation of Hepatitis C treatment to primary care settings according to comprehensive and standardised therapeutic and diagnostic protocols based on approaches similar to the public health approach to HIV care may reach more PLHCV and improve linkage to and retention in care [46]. This is anticipated to result into improved SVR outcomes, reduction of liver‐associated complications and onward HCV transmission. Decentralising HCV treatment requires the development and implementation of a national strategy to roll out the HCV treatment and strengthening of health services at lower tiers of the health pyramid.

In conclusion, achieving high cure rates with DAAs in Cameroon, a lower‐middle‐income country in sub‐Saharan Africa, is feasible. However, the accessibility and provision of HCV screening, diagnosis, treatment, monitoring and care using a simple algorithm should be addressed if treatment programmes were to be successfully implemented on a large scale.

## Supporting information


**Appendix S1.** Relevant items as included in the standardised digital case report (e‐CRF) in the CommCare application.
**Table S1.** Costs per DAA treatment regimen.
**Table S2.** Overview of laboratory examinations and costs per diagnostic.
**Table S3.** Overall Hepatitis C treatment costs calculation.
