# Supplementary material for: Achieving a high cure rate with direct‐acting antivirals for chronic Hepatitis C virus infection in Cameroon: a multi‐clinic demonstration project
Source: Trop Med Int Health. 2020 Jul 5;25(9):1098–109. doi: 10.1111/tmi.13450 (PMC7540389; doi:10.1111/tmi.13450)
Supplement: Supplementary file 1 — Appendix S1. Relevant items as included in the standardised digital case report (e‐CRF) in the CommCare application. Table S1. Costs per DAA treatment regimen. Table S2. Overview of laboratory examinations and costs per diagnostic. Table S3. Overall Hepatitis C treatment costs calculation. [file TMI-25-1098-s001.docx]

# Supplementary appendix

#### Supplement to: Coyer L, Njoya O, Njouom R, Mossus T, Kowo T, Essomba F, Boers A, Coutinho R, Ondoa P. Achieving a high cure rate with direct-acting antivirals for chronic hepatitis C virus infection in Cameroon: A multi-clinic demonstration project.

## Index

[Table S1 Costs per DAA treatment regimen 2](#_Toc31893178)

[Table S2 Overview of laboratory examinations and costs per diagnostic 3](#_Toc31893179)

[Table S3 Overall Hepatitis C treatment costs calculation 4](#_Toc31893180)

[Relevant items as included in the standardized digital case report (e-CRF) in the CommCare application 5](#_Toc31893181)

### Table S1 Costs per DAA treatment regimen

| **Treatment regimen** | **Costs in €** |
| --- | --- |
| Ledipasvir/sofosbuvir (12 weeks)  for non-cirrhotic participants with HCV genotype 1 or 4 | **477.75** (25%=119.31) |
| Ledipasvir/sofosbuvir with ribavirin (12 weeks) for cirrhotic participants with HCV genotype 1 or 4 | **568.71** (25%=142.18) |
| Sofosbuvir with ribavirin (12 weeks)  for non-cirrhotic participants with HCV genotype 2 | **491.95** (25%=122.99) |
| Sofosbuvir with ribavirin (24 weeks)  for cirrhotic participants with HCV genotype 2 | **983.90** (25%=245.98) |

### Table S2 Overview of laboratory examinations and costs per diagnostic

|  | **Follow-up moment** | | | | | | | | | **Unit price in €** | **Min and max costs for overall treatment course^#^** |
| --- | --- | --- | --- | --- | --- | --- | --- | --- | --- | --- | --- |
| **Exams** | Pre-enrolment | Enrolment | Week 4 | Week 8 | Week 12 | Week 16^†^ | Week 20^†^ | Week 24^†^ | Week 24/36^‡^ |  |  |
| Full Blood Count | x | x | x | x | x | x | x | x | x | 4.16 | 24.96-37.44 |
| ALT, AST | x |  |  |  |  |  |  |  | x | 6.24 | 12.48 |
| Albumin | x |  |  |  |  |  |  |  |  | 5.55 | 5.55 |
| Bilirubin | x |  |  |  |  |  |  |  |  | 2.08 | 2.08 |
| Creatinine | x | x^§^ | x^§^ | x^§^ | x^§^ | x^§^ | x^§^ | x^§^ | x^§^ | 1.39 | 1.39-12.51 |
| HCV plasma RNA | x |  |  |  |  |  |  |  | x | 95.33 | 190.66 |
| HCV genotyping | x |  |  |  |  |  |  |  |  | 125.65 | 125.65 |
| Ag HBs | x |  |  |  |  |  |  |  |  | 9.71 | 9.71 |
| HIV serology | x | x | x | x | x | x | x | x | x | 9.71 | 87.39 |
| HIV plasma RNA^¶^ | x |  |  |  |  |  |  |  | x | 7.62 | 0-15.24 |
| CD4 count^¶^ | x |  |  |  |  |  |  |  |  | 19.03 | 0-19.03 |
| Urine β-hCG^@^ | x | x | x | x | x | x | x | x | x | 1.52 | 0-13.68 |
| FibroTest | x |  |  |  |  |  |  |  |  | 137.94 | 137.94 |
| Liver ultrasound | x |  |  |  |  |  |  |  |  | 30.32 | 30.32 |
| ***Total*** |  |  |  |  |  |  |  |  |  |  | **628.13-699.68** |

^†^Only for patients with HCV genotype 2 and cirrhosis, who received sofosbuvir/ribavirin for 24 weeks.

^‡^Corresponding to 12 weeks post-treatment
^§^Only for HIV-positive patients receiving tenofovir

^¶^Only for HIV-positive patients on antiretroviral therapy

^@^Only for women of child-bearing age

^#^Depending on HIV status, duration of treatment course, type of DAA regimen, and probability of pregnancy

### Table S3 Overall Hepatitis C treatment costs calculation

| **Treatment items** | **Costs per patient in €** | **Comments** | **Potential for cost reduction** |
| --- | --- | --- | --- |
| Drugs  (here: branded ledipasvir/sofosbuvir for 12 weeks) | 477.75 | Price can be up to 983.90 for patients with HCV genotype 2 and receiving sofosbuvir with ribavirin for 24 weeks | Further negotiations with manufacturers and use of generic drugs |
| Full panel of diagnostic tests  (average between minimum and maximum costs) | 663.91 | Price ranges between 628.13 and 699.68 depending on HIV status, duration of treatment course, type of DAA regimen, and probability of pregnancy | Use of pan-genotypic DAA removes need for genotyping (reduction of 125.65). Use of the APRI score instead of FibroTest (reduction of 137.94). Limit the ultrasound to those triaged by the APRI score (reduction of 30.32) |
| Additional diagnostic-related costs, including vaccinations, consumables (tubes), and sample transportation | 66.43 |  | None |
| Consultations (site support costs, staff: clinicians, pharmacists, nurses) | 209.72 |  | Decrease the number of interim consultations and shift tasks to general practitioners or nurses |
| Coordination (supervision, follow-up of patients, general administration of the program) | 234.37 |  | Decentralize the treatment to reduce coordination costs |
| Monitoring and Evaluation, including electronic CRF, validation of cure rate | 253.24 |  | Reduce the control points and promote self quality assurance of the process through a pay-for-performance model |
| ***Total*** | ***1914.42***  ***(2105.71 USD)*** |  |  |

### Relevant items as included in the standardized digital case report (e-CRF) in the CommCare application

#### **Pre-enrolment visit**

#### **Date de pré-enrôlement** (date du premier contact) : (jj/mm/aaaa) I___I___I______I

#### *Par défaut la date du jour*

#### **Clinique:**

#### Hôpital central (HCY)

#### Hôpital Général (HGY)

#### Hôpital de la CNPS (ESS)

#### CHU (CHU)

#### Clinique de la cathédrale (CAT)

#### Hôpital militaire (HMY)

#### Oui Non

#### **Le patient a-t-il reçu des informations sur l’étude ?**

#### **Le patient accepte-t-il de participer à l’étude ?**

#### **La fiche de consentement éclairé a-t-elle été signée ?**

#### *S’affiche si le patient accepte de participer à l’étude*

#### **Date de signature du consentement éclairé** : (jj/mm/aaaa) I___I___I______I

#### *S’affiche si la fiche de consentement a été signée. Par défaut la date du jour*

#### ***Identification***

#### **Numéro patient:** *De 001 à 099* I__I__I__I

#### *Un contrôle est mis en place pour éviter les doublons sur la même tablette*

#### **Connaissez- vous la date de naissance du patient ?**

#### Oui Date de naissance du patient : (jj/mm/aaaa) I___I___I______I *Le patient doit être âgé de 21 ans ou plus pour entrer dans le programme. La date naissance ne peut être dans le futur.*

#### Non Entrez l’âge du patient (en année) : I___I *Seul des chiffres sont accepté. Le patient doit être âgé de 21 ans ou plus pour entrer dans le programme*

#### **Sexe :**

#### Masculin

#### Féminin

#### **Etat civil :**

#### Célibataire

#### Cohabitant(e)

#### Marié(e)

#### Veuf(ve)

#### Divorcé(e)

#### **Profession :**

#### Fonctionnaire / Agent de l’Etat

#### Employé du secteur privé

#### Travailleur indépendant

#### Sans emploi

#### Autre

#### **Lieu de résidence**

#### Ville : ___________________________________

#### Quartier : ________________________________

#### ***Entrée dans le programme* Poids du patient (en Kg)** ______________________________

#### *Le poids doit être compris entre 32 et 200kg*

#### **Taille du patient  (en cm)** ______________________________

#### *La taille doit être comprise entre 140 et 205cm*

#### **Groupe sanguine**:

#### A

#### B

#### AB

#### O

#### Ne sais pas (NSP)

#### **Facteur rhésus :**

#### *Ne s’affiche pas si le groupe sanguin est inconnu (NSP)*

#### Rh+

#### Rh-

#### **Vaccination sur l’hépatite B**(vérifier sur la base du carnet de vaccination):

#### Oui

#### Non

#### ***Antécédents***

#### Facteurs de risque de transmission:

#### Oui Non

#### **Transfusion sanguine**

#### **Chirurgie invasive**

#### **Scarifications ou tatouages**

#### (à l’examen physique ou selon les déclarations du patient)

#### **Antécédent de vaccination collective**

#### **Utilisation de drogues injectables**

#### **Risque domestique**

#### (contact avec des personnes infectées dans la vie quotidienne)

#### **Traitements traditionnels à base d’instruments tranchants**

#### **Autres facteurs de risque** : ___________________________________________________

#### **Connaissez-vous la date de la dernière transfusion sanguine ?**

#### *Ne s’affiche pas si Transfusion sanguine est « Non »*

#### Oui

#### Date de la dernière transfusion : (jj/mm/aaaa) I___I___I______I

#### Lieu de la dernière transfusion : (laissez libre si ne sait pas) _____________________

#### Non

#### Facteur de risque de sévérité :

#### **Consommation régulière d’alcool** :

#### Oui

#### Nombre de verres : ______________

#### *Ne s’affiche pas si Consommation régulière d’alcool est «  Non »*

#### Unité :

#### Par jours (/j)

#### Par semaines (/sem)

#### Non

#### **Co-morbidités** : *Contrôle mis en place pour que Aucun ne puisse pas être sélectionné avec une autre option*

#### Aucun

#### Diabète

#### Hypertension artérielle

#### Maladie mentale

#### Autre Précisez les autres facteurs de co-morbitidé : ______________

#### ***Critères d’éligibilité disponibles dans le dossier diagnostic de confirmation***

#### **Résultats du test anticorps anti VHC positifs disponible ?**

#### Oui

#### **Date du test anticorps VHC :** (mm/aaaa) I___I______I

#### **Résultat :**

#### Positif Dernières charges virales (CV) d’ARN VHC : (en copie d’ARN/ml de plasma)_

#### Date du résultat des charges virales VHC : (mm/aa) I___I______I

#### Négatif

#### Non

#### **Génotype VHC disponible?**

#### Oui

#### Génotype VHC:

#### 1a

#### 1b

#### 2

#### 3

#### 4

#### Non

#### Génotypage inclus dans le bilan de pré-inclusion ?

#### Oui

#### Non

#### **Résultats Fibrotest / Apriscore disponible** ?

#### Oui

#### Date des résultats du Fibrotest / Apriscore : (mm/aa) I ___ I ______I

#### Résultats : __________________________________________________________

#### Non

#### **Echographie standardisée faite ?** (par le radiologue assermenté de l’étude)

#### Oui

#### Date de l’échographie : (mm/aa) I___I______I

#### Résultat de l’échographie:

#### Rien à signaler

#### Cirrhose

#### Nodules

#### Autres

#### Préciser : _________________________________________

#### Non

#### **Résultats de la sérologie VIH disponible ?**

#### Oui

#### Date du test de la sérologie VIH : (mm/aa) I___I______I

#### Résultats de la sérologie VIH :

#### *Contrôle mis en place : si Sérologie indéterminée ou négative, un bandeau s’affiche pour penser à demander une Sérologie VIH*

#### *De même si aucun dépistage VIH n’est disponible.*

#### Positif

#### TAR initiée ?:

#### Oui

#### Date du début de traitement : I___I___I______I

#### Non

#### Négatif

#### Indéterminé

#### Non

#### ***Critères d’exclusion***

#### Oui Non

#### **Patient déjà sous traitement DAA ou avec**

#### **une histoire d’échec thérapeutique aux DAA ?**

#### **Taux de CD4 < 250 pendant les 6 derniers mois ?**

#### *Ne s’affiche que si Sérologie VIH positive*

#### **Patient sous TAR depuis plus de 8 semaines ?**

#### *Ne s’affiche que si Sérologie VIH positive.* *Pré rempli en fonction de la date de début de traitement*

#### **Traitement basé sur l’un des médicaments suivants :**

#### Amiodarone

#### Carbamazépine, Phénytoine, Phénobarbital, Oxacarbazépine

#### Rifabutine, Rifampine or Rifapentine

#### St John’s Wort

#### Rosuvastatine

#### **Troubles psychiatriques aigus :**

#### **Grossesse confirmée :**

#### *Ne s’affiche que s’il s’agit d’une femme non ménopausées*

#### **Allaitement en cours :**

#### *Ne s’affiche que s’il s’agit d’une femme*

#### **Patients prélevés et bilan de pré-inclusion demandé :**

#### (2 tube EDTA, 2 tube sec)

#### *Contrôle mis en place : si une des réponse est oui, un bandeau rouge s’affiche pour signifier que les critères d’inclusion ne sont pas respectés.*

#### ***Résultats de pré-inclusion***

#### **Antigène de surface hépatite B (AgHBs)**

#### Positif

#### Négatif

#### **Créatinine (mg/l) : __________________**

#### **Albumine (mg/l) : __________________**

#### **Bilirubine (mg/l) : __________________**

#### **Alate et Asat : __________________**

#### **Sérologie VIH (double test) :**

#### Positif

#### Taux de CD4 (celuules/ul de sang) : __________________

#### Charge virale VIH (cp/ml) : __________________

#### Négatif

#### Indéterminé

#### **ß-hCG urinaires** (pour les femmes en âge de procréer)

#### Positif

#### Négatif

#### ***Numération et formule sanguine***

#### Reprendre les données provenant du centre Pasteur du Cameroun

#### Globules rouges (Téra/l) __________________________

#### Hémoglobine (g/dl) ______________________________

#### Hématocrite (%) _________________________________

#### Vol glob Moy (fl) _________________________________

#### TGMH (pg) _____________________________________

#### CCMH _________________________________________

#### Globules blancs (giga/l) ____________________________

#### Neutrophiles % (pourcentage) _______________________

#### Neutrophiles (giga/l) ______________________________

#### Lymphocytes % (pourcentage) _______________________

#### Lymphocytes (giga/l) ______________________________

#### Monocytes % (pourcentage) ________________________

#### Monocytes (giga/l) _______________________________

#### Eosinophiles % (pourcentage) _______________________

#### Eosinophiles (giga/l) ______________________________

#### Basophiles % (pourcentage) _________________________

#### Basophiles (giga/l) ________________________________

#### Plaquettes (giga/l) ________________________________

#### **Enrolment visit**

#### **Date de l’enrôlement : (jj/mm/aa) I___I___I______I**

#### ***Vérification des critères d’inclusion***

#### Oui Non

#### **Le patient est-il toujours d’accords pour participer au programme ?**

#### **Test anticorps anti VHC positifs ?**

#### **Charge virale VHC positive ?**

#### **Résultats Fibrotest / Apriscore disponible ?**

#### *Les réponses aux questions 2, 3 et 4 sont complétées par défaut avec les réponses apportée dans le formulaire de pré-enrôlement*

#### *Contrôle mis en place : si l’une de ces conditions n’est pas remplie, impossible d’aller plus loin.*

#### ***Vérification des critères d’exclusion***

#### Pour être inclus, toutes les réponses aux questions doivent être non Oui Non

#### **Patient déjà sous traitement DAA ou avec**

#### **une histoire d’échec thérapeutique aux DAA ?**

#### **Taux de CD4<350 pendant les 6 derniers mois**

#### *Si patient séropositif*

#### **Patient sous TAR depuis au moins 8 semaines**

#### *Si patient séropositif*

#### **Trouble psychiatrique aigus :**

#### **Grossesse confirmée :**

#### *S’il s’agit d’une femme*

#### **Allaitement en cours**

#### *S’il s’agit d’une femme*

#### **Antécédents de cirrhose décompensée**

#### **Antécédents de Carninomes hépatocellulaire**

#### **Antécédents de transplantation du foie ou du rein**

#### **Co infection hépatite B**

#### (Si sérologie négative et patient pas vacciné, procéder à la vaccination)

#### **Insuffisance rénale**

#### **Taux d’hémoglobine < 10g/dl**

#### **Traitement basé sur l’un des médicaments suivants**

#### Amiodarone

#### Carbamazépine, Phénytoine, Phénobarbital, Oxacarbazépine

#### Rifabutine, Rifampine or Rifapentine

#### St John’s Wort

#### Rosuvastatine

#### **Voulez-vous inclure ce patient dans le programme ?**

#### ***Examen physique***

#### **Pouls (bmp) :**

#### **Tension artérielle (mmHg):**

#### *Les tensions artérielles doivent être comprises entre 50 et 220*

#### Systolique _____________________

#### Diastolique ____________________

#### *La tension Diastolique doit être inférieure à la tension systolique*

#### **Poids (en kg): _______**

#### *Le poids doit être compris entre 35 et 200 kg*

#### **Co-médication** (incluant les médicaments traditionnels) :

#### Oui Préciser la co-médication ________________________________________________

#### Non

####

#### ***Traitements***

#### **Traitement Hépatite C :**

#### *Complété par défaut en fonction du patient (génotype et cirrhose)*

#### **Option 1 :** dose fixe de Ledispavir / Sofosbuvir pendant 12 semaines

#### **Option 2 :** dose fixe de Ledispavir / Sofosbuvir pendant 12 semaines + dose de Ribavirin basée sur le poids

#### **Option 3 :** dose fixe de Sofosbuvir / Ribavirin basée sur le poids pendant 12 semaines

#### **Option 4 :** dose fixe de Sofosbuvir / Ribavirin pendant 12 semaines

#### **Traitement VIH :**

#### Combinaison Tenofovir+Lamivudine+Efavirenz

#### Combinaison Zidovudine+Lamivudine+Nevirapin

#### Combinaison Tenofovir+Lamivudine+Nevirapin

#### Combinaison Zidovudine+Lamivudine+Efavirenz

#### Combinaison Tenofovir+Lamivudine+Kaletra (Lopinavir+Ritronavir)

#### **Date du début du traitement :** (jj/mm/aa) I___I___I______I

#### *Par défaut correspond à aujourd’hui*

#### **Précisez les doses de Sofosbuvir / Rabavirin : __________**

#### *Complété par défaut en fonction du poids du patient*

#### **Ordonnance pour les médicaments donnée au patient**

#### Oui

#### Non

#### **Comptage inopiné des comprimés programmé**

#### Oui

#### Entrez la date du comptage inopiné (jj/mm/aa) I___I___I______I

#### Non

#### Si le traitement entré par l’agent est différent de celui calculé par l’application :

#### **Justification du traitement :** ____________

#### **Follow-up treatment visits**

#### **Date de la consultation** (jj/mm/aa) I___I___I______I

#### **Le patient met-il fin à sa participation ?**

#### Oui

#### Précisez les raisons de la fin de participation et *« Finish »*

#### Non

#### ***Vérification des critères d’éligibilité***

#### **Le patient est-il toujours éligible ?**

#### Oui

#### Non

#### Pourquoi pas ?

#### Grossesse

#### Séroconversion VIH

#### Autre

#### Donnez les autres raisons : _________________________________

#### ***Examen physique***

#### **Pouls (bmp) :**

#### **Tension artérielle (mmHg):**

#### *Les tensions artérielles doivent être comprises entre 50 et 220*

#### Systolique _____________________

#### Diastolique ____________________

#### *La tension Diastolique doit être inférieure à la tension systolique*

#### **Poids (en kg): _______**

#### *Le poids doit être compris entre 35 et 200 kg*

#### **Co-médication** (incluant les médicaments traditionnels) :

#### Oui Préciser la co-médication ________________________________________________

#### Non

#### ***Observance du traitement***

#### Oui Non

#### **Ce matin avez-vous oublié de prendre votre médicament ?**

#### **Depuis la dernière consultation, avez-vous été en panne**

#### **de médicament ?**

#### **Vous est-il arrivé de prendre votre traitement**

#### **en retard par rapport à l’heure habituelle ?**

#### **Vous est-il arrivé de ne pas prendre votre traitement parce que**

#### **certains jours, vous avez l’impression que votre traitement**

#### **vous fait plus de mal que de bien ?**

#### **Pensez-vous que vous avez trop de comprimés à prendre ?**

#### **Vous est-il arrive d’oublier de prendre votre traitement ?**

#### Si oui, Quelle est la raison pour laquelle vous avez oublié ? ___________

#### **Résultat comptage inopiné**

#### Nombre de comprimés = nombre anticipé

#### Nombre de comprimés > nombre anticipé

#### Nombre de comprimés < nombre anticipé

#### *Contrôle de l’observance : Si pas de oui : bandeau vert « Bonne observance » Si 1 ou 2 oui : bandeau orange « problèmes d’observance minimes » Si 3 oui ou plus : bandeau rouge « Mauvaise observance »*

#### ***Examens de laboratoires***

#### **Patients prélevés ?** (un tube EDTA, un tube sec)

#### Oui

#### Non

#### **ß-hCG urinaires :**

#### *Pour les femmes en âge de procréer*

#### Positif

#### Négatif

#### **ALT / AST** *Pour les patients cirrhotiques*

#### **Sérologie VIH :** *Pour les visites 4 et 8*

#### Positif

#### Négatif

#### Indéterminé

#### **Créatinine** (mg/l) : ______

#### *Pour les patient sous traitement VIH à base de Tenofovir*

#### **Hémoglobine** (g/dl) : _____________________

#### ***Conseils et éducation***

#### Oui Non

#### **Le (la) patient(e) a-t-il (elle) reçu les conseils**

#### **pour la prévention de la réinfection ?**

#### **Le (la) patient(e) a-t-il (elle) reçu les conseils**

#### **pour le planning familial ?**

#### **Le (la) patient(e) a-t-il (elle) rapporté des effets indésirables ?**

#### Si oui, quels sont ces effets indésirables ? ____________________________________

#### **Visit 12 weeks post-treatment**

#### **Date de la consultation** (jj/mm/aa) I___I___I______I

#### **Le patient met-il fin à sa participation ?**

#### Oui

#### Non

#### Si oui, *« Finish »*

#### ***Vérification des critères d’éligibilité***

#### **Le patient est-il toujours éligible ?**

#### Oui

#### Non

#### Pourquoi pas ?

#### Grossesse

#### Séroconversion VIH

#### Autre

#### Donnez les autres raisons : _________________________________

#### ***Examen physique***

#### **Pouls (bmp) :**

#### **Tension artérielle (mmHg):**

#### *Les tensions artérielles doivent être comprises entre 50 et 220*

#### Systolique _____________________

#### Diastolique ____________________

#### *La tension Diastolique doit être inférieure à la tension systolique*

#### **Poids (en kg): _______**

#### *Le poids doit être compris entre 35 et 200 kg*

#### **Co-médication** (incluant les médicaments traditionnels) :

#### Oui Préciser la co-médication ________________________________________________

#### Non

#### ***Examens de laboratoires***

#### **Patients prélevés ?** (un tube EDTA, un tube sec)

#### Oui

#### Non

#### **End-of-project visit**

#### **Date de la consultation** (jj/mm/aa) I___I___I______I

#### ***Examens de laboratoires***

#### **Charge virale VHC** __________________________

#### **Charge virale VIH** ______________________________

#### **ß-hCG urinaires :**

#### *Pour les femmes en âge de procréer*

#### Positif

#### Négatif

#### **ALT / AST** *Pour les patients cirrhotiques*

#### **Sérologie VIH :** *Pour les visites 4 et 8*

#### Positif

#### Négatif

#### Indéterminé

#### **Créatinine** (mg/l) : ______

#### *Pour les patient sous traitement VIH à base de Tenofovir*

#### **Hémoglobine** (g/dl) : _____________________

#### ***Numération et formule sanguine***

#### Reprendre les données provenant du centre Pasteur du Cameroun

#### Globules rouges (Téra/l) __________________________

#### Hémoglobine (g/dl) ______________________________

#### Hématocrite (%) _________________________________

#### Vol glob Moy (fl) _________________________________

#### TGMH (pg) _____________________________________

#### CCMH _________________________________________

#### Globules blancs (giga/l) ____________________________

#### Neutrophiles % (pourcentage) _______________________

#### Neutrophiles (giga/l) ______________________________

#### Lymphocytes % (pourcentage) _______________________

#### Lymphocytes (giga/l) ______________________________

#### Monocytes % (pourcentage) ________________________

#### Monocytes (giga/l) _______________________________

#### Eosinophiles % (pourcentage) _______________________

#### Eosinophiles (giga/l) ______________________________

#### Basophiles % (pourcentage) _________________________

#### Basophiles (giga/l) ________________________________

#### Plaquettes (giga/l) ________________________________

#### ***Visite de fin de programme***

#### **Suppression virale à 12 semaines post-traitement ?**

#### Oui

#### Non

#### CV rétrospective à 4 semaines demandée ?

#### Oui

#### Non

#### **Si CV post-traitement est détectable CV VIH à S12, post traitement demandée ?**

#### Oui

#### Non

#### ***Conseils et éducation***

#### Oui Non

#### **Le (la) patient(e) a-t-il (elle) reçu les conseils**

#### **pour la prévention de la réinfection ?**
